# Supplementary material for: Are carbon emissions trading and green financial instruments synergistic? -Comprehensive quantitative research based on content analysis
Source: PLoS One. 2024 Mar 7;19(3):e0298601. doi: 10.1371/journal.pone.0298601 (PMC10919601; doi:10.1371/journal.pone.0298601)
Supplement: S1 Data — (ZIP) [file pone.0298601.s001.zip › Data/地级市Z_F工作报告.zip!/╣≤╓▌/╫±╥σ╩╨/└┤╫╘╣1⁄2╓┌║┼í╛┬φ┐╦╩2╛▌═°í┐_2016.docx]

各位代表：

　　现在，我代表市人民政府向大会作工作报告，请结合《遵义市国民经济和社会发展第十三个五年规划纲要（草案）》一并审议，并请市政协委员和其他列席人员提出意见。

　　“十二五”时期工作回顾

　　过去五年，在省委、省政府和市委的坚强领导下，在市人大、市政协的监督支持下，全市上下高举发展团结奋斗旗帜，坚守发展和生态两条底线，坚定不移实施主基调主战略，经济社会发展取得显著成就，“十二五”规划的主要目标任务全面或超额完成，为“十三五”守底线走新路率先小康奠定了坚实基础。

　　“十二五”是我市经济加速发展、综合实力提升最快的五年。经济实力明显提升。初步统计，2015年完成地区生产总值2168.3亿元，比上年增长13.2%，实现五年翻番，年均增长15%；人均地区生产总值突破35000元；全社会固定资产投资3200亿元，比上年增长25.4%，五年累计完成近1万亿元，年均增长42%；一般公共预算收入177.6亿元，比上年增长11.2%，年均增长25.3%；社会消费品零售总额639.9亿元，比上年增长12.2%，增速连续五年全省第一，年均增长17.1%；年末金融机构存贷款余额分别达3306.6亿元和1925.5亿元，分别比年初增长28.8%、26%。全面小康实现程度达86.5%，红花岗、汇川、仁怀率先实现全面小康。结构调整成效明显。三次产业结构由15.4︰41.8︰42.8调整为16.1︰44.8︰39.1。工业经济达到“3个1000”新水平，完成500万元以上规模工业增加值1010亿元，比上年增长12.2%，年均增长15.6%，工业经济总量稳居全省第一；500万元以上规模工业企业达1100家，利润总额占全省的60%左右，以晴集团、海螺水泥等一批大项目建成投产；工业投资突破1000亿元，县县搭起经济开发区或工业园区平台，累计建成标准厂房933万平方米，引进786户企业入园；茶酒“姊妹篇”、装备制造等传统产业稳步发展，大数据、新能源汽车等产业从无到有，智能终端产品制造实现突破，淘汰落后产能533万吨。文化旅游提速发展，成功举办四次旅发大会，海龙屯土司遗址成功申遗，遵义会议陈列馆、土城千年古镇等一批重点旅游景区建成投用，新增级景区11个，桐梓杉坪等农旅融合景点成为休闲度假新热点，旅游接待人数、综合收入分别达6200万人次、547亿元，“红色圣地·醉美遵义”知名度美誉度大幅提升。现代服务业加快发展，遵义国际商贸城、新雪域冷链物流等一批大型综合市场建成运营，阿里巴巴·遵义产业带、淘宝·特色中国遵义馆、爱特购等电商平台相继涌现，建成一批县级电商平台，全市限额以上商贸企业达764家。农业“1234”战略和“8531”工程深入实施，茶叶、蔬菜（辣椒）、中药材、生态畜牧、干鲜果等主导产业初具规模，认证“三品一标”（1）农产品281个，粮经比由6:4调整为4:6，现代山地特色高效农业开始走向前台,省级园区达45个,市级以上龙头企业达到502家，各类农民专业合作社3310个。四大区域竞相发展。中部、西部、东部、北部四大区域生产总值年均分别增长16.7%、15.8%、15.4%和15.6%，6个县（市、区）经济总量突破100亿元，仁怀市突破500亿元进入中国百强县。县域经济比重提高到74%。

　　“十二五”是制约瓶颈快速突破、城乡面貌变化最大的五年。基础设施全面改善。遵义新舟机场通航25个城市，年旅客吞吐量突破83万人次，遵义仁怀茅台机场加快建设；建成投运仁赤、杭瑞（遵义段）、道安等一批高速公路，全市高速公路通车里程突破，如期实现“县县通高速”目标；国省干线通车里程达，是“十一五”期末的5.4倍；新增农村公路通车里程1.，基本实现村村通油（砼）路、乡乡有客运站；渝黔快铁等铁路和构皮滩翻坝运输等水运工程加快建设。开工建设骨干水源工程47座，治理病险水库186座、中小河流71条，解决328万农村人口饮水安全问题，新增供水能力，发展灌溉面积68万亩。电力总装机突破1000万千瓦，桐梓火电厂和习水二郎电厂（一期）等一批电力项目建成运行，农村电网改造基本完成。中卫—贵阳（遵义段）和遵义至习水天然气支线管道全线贯通。在全省率先实现移动信号乡乡通，与贵阳、安顺实现通信同城化。城乡建设提档升级。城镇化率提高到45%。中心城区建成区面积突破110平方公里，新蒲新区、南部新城、北部新城拉开发展格局，形成承载200万人口城市框架。“3个100平方公里工程”“八大工程”（2）“5个100民生工程”（3）深入实施，中心城区高速公路入城口全面改造，官井隧道、万里路、苏州路等“肠梗阻”全面畅通，城市微循环道路系统大为改善。所有县城建成区面积实现倍增，21个城市综合体、54个示范小城镇建设成效显著。成功创建4个国家卫生县城（城市），省级卫生县城实现全覆盖。全国文明城市创建扎实推进，9个县城（城市）成功创建省级文明城市。“四在农家·美丽乡村”创建向纵深推进，90%以上农村人口受益小康“6+1”行动计划（4），余庆县红渡等一批精品示范点点亮黔北大地。习近平总书记高度肯定“七要素”（5）黔北民居建设样板，赞誉遵义县花茂村为“找到乡愁的地方”。生态底线不断筑牢。创建国家环保模范城市通过验收；累计建成国家生态示范区5个、生态镇38个，赤水市、湄潭县在全省率先通过国家生态县（市）创建验收；营造林255万亩，治理石漠化面积753平方公里、水土流失面积1850平方公里，“月月造林”活动持续推进，森林覆盖率提高到55%；城乡生活垃圾无害化处理率达90.9%，城镇污水处理率89.9%；县城环境空气质量自动监测设施全覆盖，城市（县城）环境空气质量达标率99.2%；集中式饮用水源地水质达标率达100%，农村面源污染治理成效明显。

　　“十二五”是改革开放破立并举、发展活力动力最足的五年。深化改革蹄疾步稳。市、县政府机构改革全面完成，市级非行政许可审批事项全部取消，编制发布市级权力清单、责任清单；颁发全省第一本不动产权证书；湄潭农村改革试验区农村集体经营性建设用地入市改革走在全国前列；“四河四带”（6）建设取得实效，赤水河流域生态文明制度改革成果在全省推广；率先在全省实施公共资源交易改革；农村土地承包经营权确权登记颁证试点、医疗卫生等一大批事项改革力度加大。对外开放力度空前。遵义综合保税区筹建取得阶段性成果，遵义新舟机场临时航空口岸建成开放并开通直航香港定期航班，遵义软件园、遵义大学生创业园挂牌运行。遵义（上海）产业园建设有序推进；实施上海对口扶贫项目122个。“引金入遵”成效明显，银行业金融机构增至40家、保险机构23家、证券机构5家，与工行、交行、中国银行等签订银政合作战略协议。累计招商引资到位资金6192亿元。2015年预计外贸进出口总额突破14.85亿美元、比上年增长222.7%。创新驱动活力显现。国家创新型试点城市创建有力推进，超临界萃取技术等一批重大科研成果全国领先，建成院士工作站19个、重点实验室19家（国家级重点实验室1家），国家高新技术企业累计达93家、国家级创新型企业3家，实施国家、省部级以上科技成果转化600多项。注册商标累计达1.7万余件，其中驰名商标19件，省著名商标322件。民营经济蓬勃发展。各类市场主体突破32万户，民营经济比重提高到55%；全面落实“3个15万”扶持政策，累计发放创业担保贷款18亿元，扶持微型企业1万余户，带动就业5.76万人。

　　“十二五”是社会事业全面进步、人民群众得实惠最多的五年。城乡居民人均可支配收入分别达24997元和9249元，分别比上年增长10%、10.6%，年均分别增长10.4%、17.1%。累计减少农村贫困人口111万人，4个贫困县和111个贫困乡镇“减贫摘帽”，226个贫困村出列，贫困发生率下降到7%以下。累计新增城镇就业45万人，转移农村劳动力51万人，城镇登记失业率始终控制在4.2%以内，荣获全国创业先进城市称号。新（迁）建成各类中小学87所，新（改、扩）建中职学校14所。遵义四中、遵义县一中升格为省级一类示范性高中，遵义干部学院和4所高校近7万名师生入驻遵义大学城。医疗卫生服务体系日益完善，城乡居民大病保险全面启动，省内异地就医实现适时联网结算，市县公立医院全部实现“二甲”以上目标并取消药品加成，新农合参保率保持在98%以上。人口计生工作取得新成绩。城乡居民养老保险参保人数达310万人。新改扩建农村敬老院210所，实现乡镇全覆盖。累计实施保障性安居工程23.2万套，改造农村危房23.2万户，实施扶贫生态移民搬迁近2万户8万余人。成功创建国家公共文化服务体系示范区。以“一场四馆”（7）为重点的文化体育工程加快建设，竞技体育、群众体育快速发展，荣获2018年省十运会承办权。依法治市深入推进，“六五”普法通过验收。“平安遵义”建设成绩显著，安全生产事故起数、死亡人数连续5年实现“双降”，社会矛盾化解“余庆经验”全面推行，“三送一连心”（8）活动深入开展，社会大局保持和谐稳定，人民群众安全感和满意度跃居全省前列。食品药品安全、疾病防控、卫生监督、双拥工作、外事侨务、民族宗教、国防教育、人民防空、气象、妇女儿童、残疾人、地方志、红十字、档案等各项工作全面进步。

　　“十二五”是政府效能大幅提升、干事创业激情最高的五年。解放思想“十破十立”、以“五梳理三带头”为载体的党的群众路线教育实践活动和“三严三实”专题教育深入开展，政府自身建设和廉政建设全面加强，推动干部作风明显转变、部门服务意识明显增强、政府效能明显提升。成功举办纪念遵义会议80周年、“贵州茶一节一会”等重大活动，成功抗击“8·11”百年不遇特大自然灾害，人民群众对经济社会发展满意度位居全省前列，得到了省委“发展思路很清、发展举措很实、发展态势很好、干事创业劲头很足”的充分肯定！

　　各位代表！回首“十二五”，成绩来之不易，经验弥足珍贵。在决战决胜脱贫攻坚、率先小康的新征程中，必须坚持解放思想求创新，以善学善思善谋划的睿智，敢闯敢试敢突破的勇气，创先创优创一流的自信，走一条符合时代特征、具有遵义特色的转型发展之路。必须坚持守牢底线走新路，在全力赶超中加快转型，在转型升级中跨越发展，做到既要绿水青山又要金山银山，推动两条底线一起守、两个成果一起收。必须坚持“四轮驱动”助发展，大力推动新型工业化、新型城镇化、农业现代化、旅游产业化融合发展，实现以产为主、产城互动、产城景一体。必须坚持改革开放破瓶颈，以改革促开放，以开放倒逼改革，激发活力，增强动力，弯道取直，后发赶超。必须坚持以人为本促和谐，把保障和改善民生作为一切工作的出发点和落脚点，注重发展成果共创共享，千方百计提高人民群众生活水平和质量。必须坚持依法行政转作风，筑牢法无授权不可为、法定职责必须为的思想意识，始终在党的领导下，在法治的轨道上，依法履职，勤勉工作，用干部的辛苦指数换取群众的幸福指数！

　　各位代表！以上成绩和经验的取得，得益于党中央、国务院、省委、省政府的亲切关怀，得益于市委的坚强领导，得益于市人大、市政协的监督支持，得益于全市干部群众的艰苦奋斗，得益于社会各界的倾力相助和上海等城市的对口帮扶。在此，我谨代表市人民政府，向全市各族人民、广大干部职工，向各民主党派、工商联、人民团体、无党派人士，向驻遵解放军、武警官兵和民兵预备役人员、公安干警，向历届老领导老同志，向所有关心支持遵义经济社会发展的各界人士，表示衷心的感谢并致以崇高的敬意！

　　在充分肯定成绩的同时，我们也清醒地认识到，我市欠发达、欠开发、欠开放的阶段特征没有变，贫穷落后是主要矛盾、加快发展是根本任务的基本市情没有变，仍面临着经济总量不大、人均水平较低、产业结构不优、基础设施滞后、发展动力不强、创新活力不足，以及生态环境仍然脆弱、扶贫攻坚任务艰巨、民生改善任务繁重、人才科技短板明显等具体问题。对这些困难和问题，我们将通过艰苦细致的工作和坚持不懈的努力，认真加以解决，努力让全市人民满意！

　　“十三五”时期奋斗目标及战略任务

　　今后五年，是遵义在全省率先建成全面小康社会的决胜期，也是可以大有作为、必须奋发有为的重要战略机遇期。面对中央加大脱贫攻坚力度、强力支持革命老区开发建设这一千载难逢的历史性机遇，面对国内经济下行压力和我市新老矛盾交织、各种潜在风险并存的挑战，面对既“赶”又“转”的双重考验和全面脱贫、率先小康的双重任务，我们必须牢固树立“打破才能得生机、取直自然出新路”的理念，主动适应新常态、把握新常态、引领新常态，学习贵阳、紧跟贵阳、紧追贵阳，深度参与重庆市场竞争、与巨人同行，倾力打造开放高地、发展宝地、生态绿地、民生福地，奋力谱写好“第一个百年目标”遵义新篇章。

　　“十三五”时期经济社会发展的总体要求：高举中国特色社会主义伟大旗帜，全面贯彻党的十八大和十八届三中、四中、五中全会精神，以马列主义、毛泽东思想、邓小平理论、“三个代表”重要思想、科学发展观为指导，深入贯彻习近平总书记系列重要讲话精神，按照“五位一体”总体布局和“四个全面”战略布局（9），贯彻创新、协调、绿色、开放、共享五大发展理念，落实主基调主战略，以“坚持红色传承，推动绿色发展，奋力打造西部内陆开放新高地”为主题，以守牢底线、走好新路、率先小康为主线，以提高发展质量和效益为中心，着力实施大扶贫、大数据战略行动，走出一条有别于东部、不同于西部其他城市的发展新路，确保在全省率先全面建成小康社会、率先向现代化迈进。

　　“十三五”时期经济社会发展的预期目标：地区生产总值年均增长13%左右，突破4000亿元,占全省比重提高到25%左右,跻身西部30个非省会同类城市前5强；人均生产总值突破1万美元；三次产业结构调整为10︰46︰44；500万元以上固定资产投资年均增长20%；核算口径工业增加值年均增长13%以上；社会消费品零售总额年均增长13.5%；一般公共预算收入年均增长12%；城乡居民人均可支配收入年均分别增长13%、13.5%以上；城乡居民收入比降到2.8︰1以内。现行贫困标准下，2016年贫困乡镇全部摘帽，2017年贫困村全部出列、贫困人口全部稳定脱贫，确保全市2018年在全省率先建成全面小康社会，力争县县实现全面小康。经过五年努力，全力建设一个经济快速发展、社会协调进步、民族文化繁荣、生态优势突出、民主法治健全、人民幸福安康的美丽遵义。

　　一、强力推进大扶贫战略行动，全面夺取脱贫攻坚新胜利

　　坚持以脱贫攻坚统揽经济社会发展全局，把脱贫攻坚作为“十三五”时期的头等大事和第一民生工程，以更加解放的思想、更加坚定的信心、更加精准的举措、更加务实的作风，坚定不移实施“35669”行动计划（10），举全市之力打赢科学治贫、精准扶贫、有效脱贫攻坚战，确保如期实现脱贫攻坚“三步走”战略（11）目标，决不让贫困地区和贫困人口在全面建成小康社会中落伍掉队。全面构建大扶贫工作格局。突出“党委主责、政府主抓、干部主帮、基层主推、社会主扶、群众主体”，健全市县乡三级扶贫工作机构，实行党政主要领导“双组长”领导体制和市负总责、县抓落实、重在乡村工作机制，强化“五包”责任制（12），做到不脱贫不脱钩。全面深化沪遵合作，大力开展千企帮村、万户结对活动，动员全社会力量广泛参与，构建专项扶贫、行业扶贫、社会扶贫“三位一体”新格局。实施少数民族扶贫攻坚计划，促进少数民族贫困人口尽快脱贫。全面实施精准扶贫脱贫。强化全省“1+10”文件政策落实，重点抓好精准扶贫精准脱贫“十项行动”（13）。按照“八个精准”（14）要求，完善贫困退出机制，对贫困人口实行动态化精细化管理，做到扶贫对象和脱贫成效精准。落实目标、任务、资金和权责“四到县”，推动涉农资金有效整合，探索财政扶贫资金和农村集体资源量化到户、股份合作、入股分红、滚动发展，做到扶贫项目和资金精准。因地制宜充实配强驻村工作组，坚持“六个到村到户”（15），对两有户、两因户、两无户、两缺户（16）实施精准滴灌、靶向治疗，做到因村派人、扶贫到户精准。实行最严格的差异化考核和问责激励双重机制，建立常态化督促检查工作机制，做到考核评价和督促检查精准。按照往县城、产业园区、中心城区和重点城镇集聚搬迁的思路，全面完成18.1万人易地脱贫搬迁任务，让搬迁农民过上体面生活。全面落实贫困家庭学生和医疗救助扶贫对象资助政策，决不让寒门学子因贫失学，决不让群众因病致贫返贫。全面创新扶贫开发体制机制。针对不同区域、不同类型、不同致贫原因，因地制宜探索各具特色的脱贫致富路子，统筹抓好经济强县和非经济强县范围内贫困乡镇、贫困村的脱贫工作，探索运用大数据扶贫、典型扶贫等新途径，建设扶贫开发攻坚示范区，打造脱贫攻坚遵义模式。

　　二、强力推进产业转型升级，全面构建现代产业新体系

　　大力实施大数据战略行动，始终坚持以信息化为引领、以工业化为主导、以大数据为链条，全神贯注抓园区、凝神聚气抓工业，全力以赴守底线、凝心聚力上项目，推动信息化与一二三产业深度融合，打造茶酒、大健康与新医药、文旅农旅一体、现代制造业与现代服务业“四个姊妹篇”，形成发展新业态，打造经济新引擎。坚定不移做强新型工业。着力打造大数据、白酒、大健康、新能源节能环保、特色农产品加工“五大千亿级产业”。大力发展以大数据应用和智能终端产品制造为重点的电子信息产业，深入推进“五个计划”“六大工程”（17），加快建设智能终端产业集聚区，形成“4个1亿”产能（18），打造“遵义智造”品牌；建设大数据应用示范城市、特色服务外包产业基地和电子元器件生产基地，建成服务外包呼叫中心5万座席。完善促进白酒产业健康发展的政策措施，支持白酒企业拓展市场，着力提升“中国酱香·赤水河谷”地域品牌和全国重点名优白酒产业集聚区影响力。实施大健康医药产业发展六项计划，建设一批医药、健康养生示范园区、产业基地和企业集团。大力发展页岩气、风电、太阳能等清洁能源，开发煤层气、生物质能等新能源，着力发展节能环保低碳产业和新型建筑建材业，支持发展新能源汽车及配套产业，打造新能源汽车制造集聚区。大力发展以茶叶、特色食品、健康水等为主的特色轻工产业，做大做强一批农产品加工园区，打造农产品加工集聚区。着力培育机电制造、新材料、新型化工、烟草等百亿级产业，打造军民融合创新示范区，加快建设机电装备制造业基地。大力推动原材料、建材、化工等传统产业转型升级，建设国家新材料产业化基地。完成遵义卷烟厂异地技改。深入开展质量兴市工作，大力实施品牌带动战略。坚定不移做特山地旅游业。围绕打造国际山地旅游和度假公园目的地，推进旅游产业规模化、精品化、市场化、国际化，全力建设“全景域、四季游、多业态”山地特色旅游产品体系和精品旅游线路，加快构建“快进慢游”设施体系，全面提升旅游接待能力、服务水准和宣传营销水平，形成“全景域体验、全过程消费、全产业融合、全民化共享”旅游发展新模式，创建国家全域旅游示范区和文化旅游发展创新区。坚持以红色旅游为引领，大力发展避暑休闲、温泉度假、汽车露营、科普探险、修学旅行等新业态，加快工旅、城旅、农旅、商旅、文旅、智旅等融合发展。创建国家级景区2个以上。创新区域流域旅游资源统筹开发体制机制，着力把文化旅游业培育成千亿级战略性支柱产业。坚定不移做大现代服务业。围绕打造千亿级现代服务业目标，大力实施现代服务业“十百千”工程（19），加快发展金融保险、现代物流、研发设计、检验检测、商贸会展、软件和信息服务、中介服务等生产性服务业，推动生产性服务业向专业化和价值链高端延伸。着力发展城乡电子商务，建设一批综合性电商平台，引进培育一批电商骨干企业，打造电子商务遵义版。积极发展房地产、健康养生养老医疗、文化娱乐、社区服务、教育培训等生活性服务业，推动生活性服务业向精细化和高品质转变。坚定不移做优现代山地特色高效农业。深入实施“1234”战略，重点发展壮大茶叶、蔬菜（辣椒）、中药材、干鲜果、生态畜牧等市级五大主导产业，打造一批百亿级农业产业。继续深化农村改革，推动农业全产业链创新，发展农业先进生产力和先进生产方式，大力培育龙头企业、专业合作社、家庭农场、专业大户等新型农业经营主体，培养新型职业农民和农业经营性服务组织。抓好品种品质品牌建设，健全农产品质量安全追溯和监管体系，加大农产品“三品一标”认证力度，推进农业标准化、信息化和机械化，打造一批特色农产品品牌。大力发展循环农业，着力发展农产品精深加工，健全农产品销售、物流体系，推进农旅融合发展，实现农业“接二连三”，走出一条产出高效、产品安全、资源节约、环境友好的现代山地特色高效农业发展新路。坚定不移提升产业集聚发展水平。坚持把产业集聚和企业主体作为转型升级的主要载体和抓手，以“绿智园区”建设为引领，深入实施“1351”产业园区成长计划，建成9个100亿元级、5个300亿元级、3个500亿元级、2个1000亿元级产业集聚平台，提升产业园区、农业园区、现代物流园区、现代服务业集聚区发展质量和经营效益。深入实施工业“百千万”工程（20）和“百企引进”“千企改造”工程，落实“双培育”“双退出”行动计划（21）；培育年主营业务收入超百亿元的龙头企业、超十亿元的小巨人企业和高成长性企业。规模工业企业达1200家，限额以上商业企业达1000家，市级以上农业龙头企业达800家。民营经济比重达60%以上。培育优质企业上市。

　　三、强力推进山地特色新型城镇化，打造城乡统筹发展新格局

　　坚持以人为本，走产城景一体、铁公机联动、山水田融合、村社园统筹、文教卫配套的山地特色新型城镇化道路，构建统筹城乡发展新格局，让绿色城镇山水相融，让绿色家园美丽乡村人人向往。城镇化率达55%。着力建设黔北区域性特色城市。落实城市工作“五个统筹”（22），增强城市发展的持续性、宜居性。大力推进遵义县、绥阳县撤县设区，着力把南部新区打造成国家级高新区，形成中心城区三个国家级平台支撑、五大核心发展主战场新格局；推进仁怀、桐梓、湄潭与中心城区同城化发展。坚持疏老城、建新城，坚持“适用、经济、绿色、美观”的建筑方针，突出地方特色元素，搞好城市规划设计，加快绿色城市（23）、智慧城市、海绵城市（24）、公交都市建设，完善城市功能。构建权责明晰、服务为先、管理优化、执法规范、安全有序的城市管理体制。到2020年，全面提升200万人口区域中心城市建设品质，形成承载300万人口的遵义都市圈。着力建设新型特色城镇。在遵义县、湄潭县开展国家级整县推进新型城镇化试点。加快发展各具特色的县城。实施小城镇“十百千”行动计划（25），打造100个“六型”特色示范小城镇（26）。加快户籍人口城镇化和农业转移人口市民化，在就业创业、子女入学、社会保障、就医报销等方面实现同城同待遇。着力建设“四在农家·美丽乡村”。坚持黔北民居“七要素”要求，按照“四在农家·美丽乡村”标准体系和“三线目标”要求（27），2018年实现集中居住村寨创建全覆盖，2020年实现95%以上的农户受益。深入实施小康“6+1”行动计划，着力打造美丽宜居家园、增收致富田园、寄托乡愁故园、观光休闲公园、投资兴业乐园美丽乡村升级版，实现建设新农村向经营新农村转变。

　　四、强力推进县域经济大发展，实现区域协调发展新突破

　　大力实施县域经济“1261”行动计划（28），推动中心城区走前列、经济强县率先发展、非经济强县增比进位，确保“十三五”经济总量县县翻一番，全部过百亿。按照“中部跨越、西部转型、东部崛起、北部突破”新要求，完善促进“四大区域”差异化发展的工作思路、政策体系、领导体制和工作机制，提升区域经济竞争力。中部六县（区）大力发展大数据端产品等电子信息产业，以制药为重点的大健康医药产业，航空航天、新能源汽车等制造业、创新转型的新材料产业和电子商务、现代物流、研发设计、商贸金融、文化旅游等现代服务业，加快建设智能终端产业集聚区、现代服务业集聚区、军民融合创新示范区。西部三县（市）加快白酒、能源化工、文化旅游、健康养生产业转型升级和创新发展，着力打造特色产业创新示范区，加快建设“四河四带”，积极申报建设赤水河流域生态经济示范区。东部三县大力发展茶旅一体、休闲养生、现代优质高效特色农业、特色农产品加工等产业，加快建设“四在农家·美丽乡村”示范区、统筹城乡发展试验区、高端休闲度假养生体验区。北部三县大力实施精准扶贫精准脱贫行动和基础设施提升工程，加快发展山地特色优质高效农业，着力发展生态畜牧业、中药材、新药创制、健康养生、文化旅游等特色产业，着力建设扶贫攻坚示范区、特色农产品产销集聚区、仡佬文化旅游体验区、渝黔开放合作创新区。

　　五、强力推进生态文明建设，加快建设绿色遵义新家园

　　坚持绿色发展，构建资源节约型、环境友好型社会，让青山更多彩、碧水更长流、土壤更洁净、天空更蔚蓝，推动人与自然和谐共生。扎实推进主体功能区规划编制。完善相关配套政策，推动优化开发区、重点开发区、农产品主产区、重点生态功能区按主体功能发展。推进“多规合一”，实现一个市县一本规划、一张蓝图。加快中心城区饮用水源调整和保护，推进赤水河、乌江、洛安江、芙蓉江等流域生态文明先行示范区建设。扎实推进生态建设。落实“绿色贵州”建设三年行动计划、地质灾害三年综合治理行动计划和林业产业三年倍增计划，实现25度以上坡耕地全部退耕还林还草，森林覆盖率提高到62%。实施石漠化、水土流失、中小河流综合治理等生态建设工程，建成国家生态文明建设示范市。扎实推进环境治理。深入实施大气、水、固体废弃物、土壤污染防治行动计划，实施重点流域、区域、行业整治，推进农村面源污染防治，加强环保基础设施建设，实现全市城乡污水处理厂和垃圾收运系统全覆盖，城乡生活垃圾无害化处理率达94%，城镇污水处理率达95%，中心城区环境空气质量达标率达90%以上，集中式饮用水源地水质达标率达100%。创新环保设施建设运营管理模式，推进环卫作业市场化，形成政府、市场、公众多元共治的环境治理体系。扎实推进循环低碳发展。建设全国新能源示范城市、国家低碳试点城市。积极倡导绿色生产生活方式，实施循环发展引领计划、全民节能行动计划，实施最严格的能源和水资源消耗、建设用地等总量和强度双控行动。单位生产总值能耗下降12.5%，万元GDP用水量下降到，建设节约型社会。推动以循环工业、循环农业、循环园区为主的循环经济发展，实施100个循环经济示范项目，打造10个循环经济示范区，形成10条循环经济产业链，促进生态产业化、产业生态化。扎实推进生态文明制度改革。总结推广赤水河流域生态文明制度改革成果，落实最严格的环境保护制度，建立全市生态文明建设考评机制、自然资源权属登记和用途管制制度。建成实时在线环境监控系统。完善环境保护惩防并举的行政管理体系，以零容忍态度打击环境违法行为。

　　六、强力推进基础设施大会战，实现发展支撑条件新提升

　　坚持基础设施建设先行，加快形成适度超前、功能完善、配套协调、互联互通的基础设施网络体系。坚持大交通引领大发展。建设现代综合立体交通网络。建成渝黔快铁、川黔铁路遵义城区段线路外迁工程、城市轨道交通1、2、3号线，开工建设攀昭黔、遵义至泸州铁路和开阳至遵义城际轨道交通，加快涪陵至柳州、贵阳经襄阳至郑州客专等铁路前期工作，确保全市铁路营业里程达到，城市轨道交通里程达；加快贵遵复线、遵崇复线、绥正、南环、习正、余遵、务彭、遵仁、道武、湄潭至新舟机场等高速公路建设，推进国省干线公路升级改造，加快建成便捷高效的骨干公路路网，建成一批“多式零距离换乘”交通枢纽；实施农村公路建设三年行动计划，建设农村公路1.67万公里，打通农村公路最后；加快综合交通运输枢纽和站场建设，实现村村通客运目标；完成遵义新舟机场改扩建，建成遵义仁怀茅台机场，争取建设黔东北机场，组建通用航空公司，力争建成3个以上通用机场；推进出省水运通道建设，提高乌江、赤水河通航能力。加快完善城乡用水保障体系。加大骨干水源、供水管网和民生水利保障工程建设；开工建设观音水库大型水利枢纽工程及45座中小型水库，续建31座中小型水库，新增供水能力，基本解决工程性缺水问题；实施农村饮水安全巩固提升工程，农村饮用水集中式供水人口比例达90％以上；力争开工建设仁赤习等大型灌区续建配套和节水改造工程，发展灌溉面积60万亩。加快信息和能源基础设施建设。实施信息基础设施建设三年行动计划和“无限网络·满格遵义”“宽带乡村”“景区景点免费WIFI覆盖”项目建设，推进骨干网络优化、城乡网络全覆盖、农村宽带延伸等重大信息基础设施建设，加速“三网融合”，形成“出市宽、市内联、覆盖广、资费低”的信息设施体系。加强输电通道建设，完善500千伏骨干电网，加快建设220千伏电网和农村电网升级改造，构建安全、稳定、合理、适度超前的区域供电网络。规划布局“一主一环五支”（29）输气管道，加快建设县城区、园区生产生活管网，实现县县通天然气管道。

　　七、全面推进改革开放创新，打造西部内陆开放新高地

　　坚持以开放倒逼改革，以改革促进创新，以创新推动发展。全面深化改革。按照中央、省委和市委全面深化改革部署，全面完成各项改革清单任务，在对外开放、行政审批、国有企业、资源配置、财税金融、投融资、电力、生态文明建设、脱贫攻坚、社会事业、法治建设等领域，集中推出一批力度大、措施实、接地气的改革方案。扎实抓好湄潭农村改革试验区等国家改革试点，新增一批国家级、省级改革试验试点，力争在打造西部内陆开放新高地等重点领域和关键环节改革上取得决定性成果。全面扩大开放。牢固树立全域开放理念，以建设开放型城市为引领，大力打通开放通道，全力全面联通并提升进入成渝地区的交通、通讯基础设施,向北融入重庆和丝绸之路经济带，向东依托重庆国家一类开放水运口岸进入长江经济带，向南联结泛珠三角经济区并延伸至东盟，向西联结“中国-中南半岛”经济走廊（30），规划建设渝黔快铁经济带。加强与新加坡、韩国、瑞士、港澳台等国家和地区合作。全面深化渝遵、沪遵合作，促进产业园区共建双赢，力争让遵义成为上海自贸区的一块“飞地”。全力申建遵义新舟机场正式航空口岸，建成遵义综合保税区，打造空港经济区和外贸出口基地，培育壮大外贸经营主体。努力建成遵义国家级高新技术产业开发区，提升遵义国家级经济技术开发区、遵义软件园、遵义大学生创业园建设营运水平，做实做强“六大开放平台”（31）。着力优化开放环境，加大招商引资和招才引智引技力度，引进优强企业和引领性大项目。大力发展总部经济和会展经济，高水准办好“贵州茶一节一会”、省市旅游产业发展大会，全力打造2个以上国际性展会平台。支持民营经济加快发展，培育“遵商精神”，打造“遵商联盟大会”品牌。全市外贸进出口总额、招商引资到位资金年均分别增长20%和15%以上。全面推动创新。围绕“5个100工程”等重要平台，突出企业创新主体地位，深入开展科技创新“五大行动计划”（32），重点推进科技、管理、产品、业态等创新，加强市药品与食品开发公共服务平台等科技创新平台建设，加大科技创新投入，设立科技创新基金，健全产学研用合作机制，积极申报组建国家重点实验室和国家工程技术研究中心，大力发展科技服务业。创新现代金融管理体制机制，积极发展互联网金融。创新人才发展机制，加快创新人才队伍建设，落实“百千万”人才引进计划（33）和“15851”人才精英工程（34），每万人人才数达1300人以上。强化知识产权保护，提高科研人员成果转化收益分享比例。新增发明专利5000件以上，综合科技进步水平指数达到68%，进入国家创新型城市行列。

　　八、强力推进民生共建共享，不断满足人民生活新期待

　　坚持人人参与、人人尽力、人人共享，千方百计保障和改善民生，让改革发展成果更多更公平更实在地惠及广大人民群众。更加突出教育为先。全面实现“新两基”（35）。优化城乡教育资源配置，有效解决中心城区、县城大班额问题，推进县域内义务教育基本均衡，促进高中教育特色发展、职业教育产教融合、高等教育内涵式发展，积极发展特殊教育、民族教育和继续教育，支持和规范民办教育，基本实现教育现代化。全面提升遵义大学城建设和管理水平，加强高校创新能力建设，确保成功申建遵义大学，力争成功申建遵义医科大学，申报遵义医专本科层次高等院校，建成贵州航天职院新蒲校区，推动遵义职院转型升级。把遵义干部学院打造成全国有影响力的干部教育培训基地。持续实施各类教育工程，优化教育办学条件。大力实施“名师名校长”工程和教育领军人才培养计划。更加突出就业为本。大力推进“大众创业、万众创新”，坚持众创、众包（36）、众扶、众筹（37），实施全民创业行动计划，增大产业对劳动力的吸附能力，实施“技能兴遵”培训项目，全面促进易地扶贫搬迁移民、返乡农民工、高校毕业生、城镇困难人员、退役军人等重点群体就业创业，发挥小微企业就业主渠道作用，城镇登记失业率控制在4.2%以内。更加突出健康为基。全力打造健康遵义，以全民健康助推全面小康。着力实施医疗卫生“五大工程”（38），实现医疗卫生资源总量持续增加、医疗服务环境明显改善、医疗卫生体制改革明显见效、群众医疗负担明显减轻、人民健康水平明显提升以及医疗卫生服务的公平性、可及性和满意度明显增强“六大目标”。发挥市第一人民医院的龙头作用，实现市县乡远程诊疗网络全覆盖。积极应对人口老龄化，加强人口和计生工作，落实全面两孩政策。积极筹建市级体育产业集团，全力办好省十运会。促进群众体育和竞技体育加快发展，实现“一场四馆”县县全覆盖和体育健身工程镇村全覆盖。更加突出文化为魂。围绕建设文化强市，提升遵义文化软实力目标，坚持红色文化传承，着力打造红色文化“4个1”工程（39），弘扬长征文化红色基因和地域历史文化、仡佬民族文化等优秀传统文化；加强思想道德建设，举办“遵道行义”大讲堂，构筑“遵道行义·自强不息”城市精神体系；倡导全民阅读，共创“书香遵义”；加强国家公共文化服务体系示范区后续建设，实施文化精品工程和“513”文化产业工程（40），振兴“遵义少儿杂技”等地域传统文化品牌；支持茅台申报世界“双遗产”（41）、双河洞申报世界自然遗产；加强市县档案馆建设；推进诚信社会建设；确保2017年成功创建全国文明城市。更加突出社保为重。落实全民参保计划，建成覆盖城乡居民的社会保障体系，加快社会保险关系跨地区转移接续。推进城乡居民养老保险制度建设。落实统一的城乡居民基本医疗保险制度。高度重视城市贫困人口帮扶工作。健全老年人、未成年人和残疾人等特殊群体关爱服务体系。抓好社会救助，积极发展慈善事业。实施保障性安居工程，全面完成易地扶贫搬迁和农村危房改造任务，大力推进棚户区改造和城镇保障性住房建设，实现住有所居。更加突出社会治理为要。深化“平安遵义”建设，建好“七张网”（42），大力实施“六项工程”（43），创新完善立体化社会治安防控体系，不断提高群众安全感和满意度。全面开展“七五”普法，持续深入开展“三送一连心”活动和法律援助工作。加强“双拥”工作，力争蝉联“全国双拥模范城”。实施“数据铁笼”计划（44），推行大数据、网格化服务管理，提升政府科学决策和风险防范水平。深入推进“四民社区”建设和民族团结进步创建活动。创新群众工作机制，把信访纳入法治化轨道，及时就地解决群众合理诉求。要采取更坚决的措施，全方位强化市场监管和安全生产，全过程保障食品药品安全。

　　各位代表！“十三五”的美好愿景令人期待、催人奋进。我们将切实增强践行“三严三实”的自觉性，以全新视野把握新形势，以全新理念引领新发展，以全新实践走出新路径，以全新目标开创新局面。我们将全面推进法治政府建设，依法履行政府职能，提高依法行政水平；我们将进一步简政放权、放管结合、优化服务，坚决取消一切不必要的行政审批，大力推进政务公开，打造全覆盖、全方位、全联通、全过程、全天候公共服务网络平台，严肃查处不作为、慢作为、乱作为行为，自觉接受各方监督；我们将始终保持定力、精准发力，一张蓝图绘到底，一以贯之干到底，切实提高政府的执行力、公信力，把一幅幅宏伟蓝图变成生动实践，把一个个规划目标变成美好现实，决不辜负全市人民的殷切期望！

　　2016年主要目标任务

　　今年是实施“十三五”规划的开局之年，抓好今年工作，对于实现后发赶超、率先小康至关重要。我们将按照市委关于今年经济工作的总体要求，坚持把“五大发展”理念贯穿经济社会发展始终，坚持稳中求进工作总基调，坚持力推创新，更加注重推进结构性改革、更加注重中央“五大政策”（45）落地，千方百计推进“五大任务”（46）落实，奋力实现“十三五”时期经济社会发展良好开局。

　　主要预期目标：地区生产总值增长13%；规模工业增加值增长12.5%，力争13.5%；500万元以上固定资产投资确保增长20%，力争25%；社会消费品零售总额增长13.5%；一般公共预算收入确保增长10%，力争12%以上；城乡居民人均可支配收入分别增长13%、13.5%；居民消费价格指数控制在省调控目标范围内，节能减排、安全生产等指标控制在省下达目标内；新增城镇就业12.6万人，转移农村劳动力10.5万人；全面小康实现程度达90%，赤水市、遵义县全面小康达标。

　　第一，着力抓好脱贫攻坚。完成全部贫困乡镇摘帽、300个贫困村出列、21万贫困人口脱贫。扎实抓好遍访贫困村贫困户和“千企帮村、万户结对”活动，大力实施“五个一批”（47）工程。突出打好易地脱贫搬迁关键一仗，注重同解决“三个1亿人”（48）问题、去房地产库存、发展产业园区和职业教育等方面结合起来，探索创新多种易地脱贫搬迁模式。把扶贫攻坚纳入全市项目观摩重要内容。较大幅度增加专项扶贫资金规模，支持每县发展1-2个主导产业，确保产业和资金覆盖50%的贫困乡镇、70%的贫困村和80%的贫困人口。切实落实贫困家庭学生教育资助政策，把贫困人口全部纳入重特大疾病救助范围。办好新型农民夜校，加强对贫困人口的技术、技能培训。高质量、高效率抓好上海对口帮扶项目，在实施遵义承接上海对口帮扶“十三五”规划上开好局、起好步。

　　第二，着力保持经济平稳较快增长。大力开展项目建设年活动。抓好国家专项建设基金争取和落地，创新运用产业基金、PPP（49）、众筹等投融资新模式，优化投资结构，加快培育新的发展动能。新开工建设市级以上重大工程项目350个以上，完成500万元以上固定资产投资2100亿元，其中工业投资620亿元、城建投资1040亿元、交通投资320亿元、农村基础设施投资40亿元、水利投资50亿元、社会事业和其它投资30亿元。大力开展市场促销年活动。积极引导企业加大产品创新和营销力度，帮助企业拓宽销售渠道。推动县县建立农村电子商务平台，重点培育5-10家本地电商企业。改造县乡农贸市场20个以上。精心筹办各类大型促销活动，推动以消费新热点、消费新模式为主要内容的消费升级。大力开展外贸突破年活动，加大对外贸龙头企业的培育和扶持力度，新增外贸企业50家以上，新增外资企业10家以上，实际利用外资增长28%以上。支持茶酒、电子信息等重点外贸企业开拓境外市场，外贸进出口总额增长20%以上。大力开展精准招商年活动。强化点对点招商、产业链招商、驻外招商分局招商，瞄准重庆先进制造、电子信息等主导产业，开展合作招商；瞄准北京、上海、广州、深圳等地的一流高科技企业或高技术含量、高科技创新成长型企业抓好精准招商，提高招商的成功率。力争引进国内外500强企业和行业龙头企业20个以上，招商引资到位资金增长15%。

　　第三，着力推进经济结构调整。工业转型升级。推进“绿智园区”建设，深入实施工业“百千万”工程，开工3000万元以上产业项目500个，建成投产120个以上；稳步提升白酒产业，发展线上线下白酒经营、交易、储存新业态，助推白酒产业调整升级；制定实施“互联网+”行动计划和年度目标计划，实施一批“互联网+”重大项目，全面推进智能终端产业集聚区建设，建成投运服务外包呼叫中心5000座席，信息产业规模达800亿元以上；推动页岩气等新能源开发利用，加快巴斯巴新能源汽车等重点项目建设；落实促进大健康医药产业发展的政策措施，加快红花岗、道真、新蒲等大健康医药产业园建设；推动能源、原材料等传统产业、国有和军工企业运用互联网技术创新生产方式、管理模式和生产工艺，实现转型创新发展；推动中心城区老工业企业改制重组和整体搬迁；建立开发区“管委会+经营公司”和“政府委托、园区办理、部门备案”运作模式，加快完善园区路网、电力、通信等基础设施，强化园区产业配套，促进产业园区转型升级。山地旅游业提标加速。以承办全省第十一届旅发大会为契机，落实好省支持我市旅游业发展的政策措施，推动全景域旅游加速发展；创建国家全域旅游示范区，加快推进24个省级重点旅游景区建设，建成赤水河谷旅游公路、湄凤余茶区木栈道等160个重点项目，启动全景域长征文化体验园和红色旅游主题公园建设，接待游客人次和旅游综合收入分别增长35%以上。现代服务业走出洼地。加快互联网金融、电子商务、房地产、现代物流、服务外包、居家养老、文化体育、住宿餐饮等生产性、生活性服务业发展，开工建设黔北现代物流新城，大力推进省级电子商务示范基地（企业）建设。农业提质增效。主动引进先进生产力、生产方式进入农业领域，提高农业现代化水平。加强现代高效农业园区建设，创建一批农旅一体化示范点；大力抓好无公害、绿色、有机农产品规模化生产，促进品牌建设、市场销售和质量监管，让群众吃得放心；高水准办好“贵州茶一节一会”，推进“东有龙井、西有凤冈”深度合作；实现农业增加值增长6%、粮食产量260万吨以上，收购烟叶140.5万担，建成城市保供蔬菜基地10万亩。培育企业主体。大力发展总部经济，启动实施“百企引进”“千企改造”工程，坚持内外资企业、大中小微企业、工农商企业一视同仁，满腔热情为企业排忧解难，帮助企业降低成本，把企业家举过头顶，全力营造重商、亲商、敬商、扶商发展环境，推动具备条件的企业上市；新增规模以上工业企业100家以上、限额以上商业企业100家、市级以上农业龙头企业50家。

　　第四，着力加大城乡建设力度。加快中心城区、县城和21个城市综合体、56个示范小城镇建设，加快户籍制度改革和居住证制度双落地，推进农民工市民化，户籍人口和常住人口城镇化率分别提高到38%和47%。加快推进“多规合一”，加速完善新蒲新区城市功能，强化北部新城产城互动，创新南部新区管理体制机制并强力推进开发建设。加快推进遵龙大道、高新快线延长线、凤朝门至新蒲城市快速通道、绥阳至新蒲同城大道和西安路建设，建成大连路与长沙路立交桥等工程，推动新老城区互联互通。力争开工建设城市轨道交通1号、3号线，开工建设遵义东站高铁新城、新蒲新区地下综合管廊，强化城市道路交通、环卫设施、停车场站等公共设施的建设和管理。加快智慧城市建设，建成“12345”市民热线。改革城市管理体制，推进城市综合执法。扩大“四民社区·美丽城市”创建覆盖面，提升全国文明城市创建水平。确保国家卫生城市通过复审和遵义县、桐梓县、绥阳县成功创建国家卫生县城。制定“四在农家·美丽乡村”建设全覆盖规划，继续实施小康6+1行动计划，在面上纵深拓展的同时，建成升级型、特色型示范点30个以上。

　　第五，着力推动生态文明建设。全面开展生态文明建设示范市创建。力争新增4个省级生态县（市、区）和54个国家级、59个省级生态乡镇。扎实开展“月月造林活动”，完成营造林75万亩以上，森林覆盖率提高到57%。建成20个山体公园、10个以上省级湿地公园。治理水土流失面积87平方公里、石漠化面积179.5平方公里。抓好乌江、芙蓉江流域污染治理和生态环境保护，加大对中桥水库周边及上游污染的治理力度。深入开展“六个一律”环保“利剑”专项行动和“六个严禁”保护森林资源专项行动，建成中心城区环境空气质量预警系统。中心城区空气质量优良率达85%以上、污水处理率达95%以上、生活垃圾无害化处理率达100%；城镇污水处理率达93%以上，县城生活垃圾无害化处理率达93.5%以上，集中式饮用水源地水质达标率达100%。启动赤水河流域生态经济示范区建设，举办赤水河流域生态经济高峰论坛。抓好洛安江流域生态文明先行示范区建设。推进国家新能源示范城市、国家低碳城市创建，抓好节能减排；工业固体废弃物综合处置利用率达90 %以上。推动红花岗区省级节水型社会建设试点和绥阳、湄潭省级水生态文明试点县建设。

　　第六，着力突破基础设施建设。抓好交通设施建设。开工建设遵义新舟机场改扩建二期工程，实现遵义仁怀茅台机场建成通航，启动通用航空机场建设前期工作；完成渝黔快铁、川黔铁路遵义城区段线路外迁工程年度建设任务；开工建设遵义至仁怀（复线）、遵义至余庆、湄潭至新舟机场高速公路，扎实推进江习古（遵义段）、贵遵（复线）、绥正、习正、南环等7条高速公路建设；加快习酒厂至茅台、旺草至青杠塘等国省干线改造；开工建设农村公路以上，全面实现村村通油（砼）路，建制村通客运率达90%以上；加快颜村综合客运站建设和忠庄客运站搬迁，启动南白客运枢纽及习水、正安2个县级客运站建设；加快构皮滩过船设施工程建设，完成湄江航运建设工程。抓好水利设施建设。力争开工建设观音大型水利枢纽工程，开工建设9座骨干水源工程，加快遵义绿塘、正安新洲、道真沙坝加高等31座中小型水库建设和南郊水库取水点上移前期工作。抓好能源通讯设施建设。新建10千伏线路、低压线路，加快官庄、角木塘等重点水电站工程和正安天楼山等3个风电场建设，积极推进有条件的地方加快天然气管道建设。深入推进信息基础设施三年会战，乡镇以上区域网络基本实现全覆盖，农村有效区域覆盖率达到80%。

　　第七，着力增强发展动力活力。深化重点领域改革。认真承接落实中央推进供给侧结构性改革政策措施，紧紧围绕打造西部内陆开放新高地，深入推进对外开放、行政管理、开发区（工业园区）、国有企业、财税金融、资源配置、生态文明建设、统筹城乡发展、旅游综合、科技、扶贫、消费、社会治理和民生领域等体制机制改革，全面推进农村土地承包经营权确权登记颁证工作；深化商事制度改革，落实支持大众创业、万众创新政策措施，激发加快发展的内生动力。加强开放平台建设。完善推进“六大开放平台”建设的措施，力争遵义综合保税区获得国家批准、遵义新舟机场正式航空口岸进入国家口岸建设“十三五”规划；完成遵义国家级经济技术开发区总体规划修编，加大扩区移位争取力度；加快申报遵义国家级高新技术产业开发区前期工作；借助遵义新舟机场直飞香港和“贵州茶一节一会”、正安·国际吉他文化艺术节等国际性节会平台，推动与新加坡、瑞士、韩国等国家和港澳台地区交流合作取得新进展；深化对重庆的认识，依托地缘优势，向重庆靠近，与重庆同行；加强沪遵两地在文化教育、医疗卫生、园区建设、科技创新、智力帮扶等领域的交流合作。着力推进创新发展。深入实施科技创新“五大行动”，推进国家创新型试点城市建设；加快新蒲高新产业园区和娄山关高新区发展。全年新增市级创新平台20家，省级科技型企业50家，申请发明专利1000件以上。着力活跃区域经济。支持各地特色优势产业发展，加强城乡建设，做实发展平台，确保绥阳县、赤水市经济总量突破100亿元，全市百亿级县（市、区）达8个。加强“四大区域”精准调度，推动地区生产总值中部增长14%、西部增长13.5%、东部增长14.5%、北部增长15%以上。

　　第八，着力防范化解各类风险。加强经济风险防控。支持原材料产业转型升级发展。实施城镇住房制度改革，提高棚改货币化安置比例，积极消化房地产存量。帮助民营企业、中小企业解决融资难、融资贵等问题。加强金融风险防控。强化对金融机构监管，切实抓好源头预防；推进社会诚信体系和金融生态环境建设，严厉打击非法集资行为。加强债务风险防控。进一步规范政府性债务管理，建立政府性债务风险综合管控机制，切实防范和化解债务风险。加强财政资金绩效管理，提高资金使用效率。加强社会风险防控。落实安全生产综合监管措施，突出重点领域和行业的安全隐患排查治理，尤其要高度重视城市安全工作和地质灾害防范治理。加大对“两违”建设行为的打击和预防力度。完善应急救援体系，坚决遏制重特大安全生产事故发生；严格维稳责任，做好重大事项社会稳定风险评估；依法严厉打击各类犯罪，坚持多渠道多角度多方式化解矛盾纠纷，普遍建立网格化管理、组团式服务、片组户联系制度，从源头上化解社会矛盾，确保群众安全感和满意度保持在全省前列。

　　第九，着力办好十件民生实事。一是完成易地扶贫搬迁7.3万人。二是完成通村道路硬化618条。三是实施60万人农村安全饮水及巩固提升工程，重点解决31个贫困乡镇、871个贫困村、10万贫困人口饮水安全问题。四是完成100所农村学校“全面薄改”（50），新建、改扩建150个乡镇（街道）及村级幼儿园。五是建成市第一人民医院内科综合大楼，完成20个乡镇卫生院达标工程，建成200家村卫生室。六是建成市水上项目训练基地、市体育健身中心游泳馆、遵义文化馆、遵义奥体中心。七是实施保障性安居工程10万户以上，改造农村危房5万户。八是实施中心城区小街小巷垃圾集中收运系统工程、亮化及路灯升级改造工程、主次干道内涝治理工程、小微循环路网二期改造工程和100个社区排污管网二期改造工程。九是全面完成中心城区集中式饮用水源地和赤水河流域乡镇污水处理设施建设。十是新建成通信基站(设备)4700个（套）以上；改造城市光纤入户端口32万户；实施223个小区宽带提速工程，覆盖住户11.24万户。

　　第十，着力提升政府服务效能。按照“三严三实”要求，加强政府效能建设，强化扣扣子、担担子、钉钉子意识，当高效率的公务人员，建高效率的人民政府。全面推进工作创新、服务创新、路径创新、模式创新、制度创新，切实增强创新能力。坚持依法行政，决不法外设权，所有行政决策执行都要于法有据；加强行政复议工作，提高行政复议效率。加快公共服务平台建设，做到应进必进、充分授权。加大市级行政审批权限下放后的服务、监督力度，做到接得住、用得好。大力推行负面清单（51）管理，落实政府权力清单、责任清单。加大政府向社会力量购买公共服务力度。推进市直机关重点岗位科级干部轮岗交流。强化行政监察、审计监督、督促检查、媒体监督、行政问责等措施，严肃查办破坏发展环境、损害企业和群众利益的行为；认真落实党风廉政建设责任制，强化政府廉政建设，加强对重点领域、重要岗位、关键环节风险防范，推进政府廉政建设和反腐败工作取得新成效，大力营造清廉、务实、担当的政务环境。

　　各位代表！美丽遵义新蓝图已经绘就，跨越赶超新征程扬帆起航。让我们紧密团结在以习近平同志为总书记的党中央周围，在市委的正确领导下，同心同德、同频共振、同甘共苦，鼓起劲来抓发展，弯下腰来拔穷根，携起手来奔小康，为实现遵义经济社会发展历史性跨越而奋勇前进！
